# Chemical and Biological Characterization of Metabolites from *Silene viridiflora* Using Mass Spectrometric and Cell-Based Assays

**DOI:** 10.3390/biom14101285

**Published:** 2024-10-11

**Authors:** Nilufar Z. Mamadalieva, Alexey Koval, Maksud M. Dusmuratov, Hidayat Hussain, Vladimir L. Katanaev

**Affiliations:** 1 Institute of the Chemistry of Plant Substances, Uzbekistan Academy of Sciences, Mirzo Ulugbek Str. 77, Tashkent 100170, Uzbekistan; 2 Tashkent Institute of Irrigation and Agricultural Mechanization Engineers, National Research University, Kori Niyazov Str. 39, Tashkent 100000, Uzbekistan; 3 Department of Pharmacy and Chemistry, Faculty of Medicine, Alfraganus University, Tashkent 100190, Uzbekistan; 4 Translational Research Center in Oncohaematology, Department of Cell Physiology and Metabolism, Faculty of Medicine, University of Geneva, 1211 Geneva, Switzerland; alexey.koval@unige.ch; 5 Department of Organic Synthesis, Faculty of Industrial Pharmacy, Tashkent Pharmaceutical Institute, Oybek Str. 45, Tashkent 100015, Uzbekistan; maqsudmansurovich17@gmail.com; 6 International Joint Laboratory of Medicinal Food Development and Health Products Creation, Biological Engineering Technology Innovation Center of Shandong Province, Heze Branch of Qilu University of Technology (Shandong Academy of Sciences), Heze 274000, China; hussainchem3@gmail.com

**Keywords:** *Silene viridiflora*, UHPLC-MS, metabolite annotation, cytotoxicity, TNBC, Wnt signaling

## Abstract

A comprehensive metabolite profiling of the medicinal plant *Silene viridiflora* using an UHPLC-ESI-MS/MS method is described for the first time. A total of 71 compounds were identified and annotated, the most common of which were flavonoids, triterpene glycosides, and ecdysteroids. The three major compounds schaftoside, 26-hydroxyecdysone, and silviridoside can be chosen as the markers for the assessment of the quality of *S. viridiflora* preparations. The methanol extract and a variety of metabolites identified in *S. viridiflora* were screened for their cytotoxic and Wnt pathway-inhibiting activities against triple-negative breast cancer (TNBC), the deadliest form of cancer in women. 2-Deoxy-20-hydroxyecdysone with submicromolar IC_50_ was identified as a result. The structure–activity relationship derived from the data from the in vitro proliferation assay showed that the hydroxyl group present at position C-2 of steroid core reduces the ecdysteroids’ cytotoxicity against cancer cells.

## 1. Introduction

Caryophyllaceae is a large family of flowering plants commonly known as the pink family or carnation family. It includes around 80 genera and more than 2600 species, distributed worldwide. Species within this family offer a diverse range of uses across ornamental, medicinal, culinary, ecological, and cultural contexts, making it an important group of plants with practical significance [1]. *Silene* L. is a large genus of plants from the Caryophyllaceae family with more than 700 species of annual, biennial, and perennial plants. Young shoots and the leaves of some *Silene* species are used as medicine and food in European countries. *Silene viridiflora*, a member of the Caryophyllaceae family, is native to Eurasia and widely distributed in Crimea–Siberia and Mediterranean countries (France, Italy, and the Balkans) [2]. It is a perennial and grows primarily in temperate biomes. The plant has simple, broad leaves and capsule fruits. Individuals can grow to 50–80 cm. The aerial parts of *S. viridiflora* contain ecdysteroids (up to 1.5% dry basis), triterpene glycosides, lipids, neutral substances, carbohydrates, and microelements [3]. Biological activities, such as tonic, immunomodulator, actoprotector, adaptogen, antioxidant, and enzyme inhibitory activities, have been reported for the extracts, ecdysteroids and mixtures of ecdysteroids isolated from the *S. viridiflora* [3,4,5,6]. Ecdysteroids and triterpene glycosides are regarded as a common phytochemical feature of *S. viridiflora* [3,7,8]. Nevertheless, no data are present on the whole metabolite composition in the aerial parts of this species. Therefore, it was relevant to conduct a phytochemical analysis of *S. viridiflora* in order to understand the chemistry for the potential health benefits of this plant.

In the present work, the ultra-high performance liquid chromatography–electrospray ionization–tandem mass spectrometry (UHPLC-ESI-MS) was applied to investigate the metabolite composition of the methanolic extract obtained from the aerial parts of *S. viridiflora*. In addition, the methanol extract and a variety of metabolites identified in *S. viridiflora* were screened for their potential to influence Wnt signaling and proliferation in triple-negative breast cancer (TNBC) cells. As TNBC is the deadliest form of gynecological cancer and is the target of massive attempts to develop natural-product-based anticancer compounds [9,10,11], such information is notably essential to obtain a deeper insight into the bioactive molecules in *S. viridiflora* and their mechanisms of action. A structure–activity relationship (SAR) study then determined the main structural features required to inhibit TNBC cells for the tested set of ecdysteroids.

## 2. Materials and Methods

### 2.1. Chemicals and Reagents

Methanol, acetonitrile, water and formic acid were purchased from Merck (Darmastadt, Germany). Ultrapure water and ammonium formate (≥99%, MS eluent additive) were obtained from Sigma Aldrich (Steinheim, Germany). Docetaxel was purchased from LC Laboratories (Woburn, MA, USA). The reference compounds quinic acid, *p*-coumaric acid, ferulic acid, oleanolic acid, isovitexin-7-*O*-glucoside, and rutin were purchased from Sigma-Aldrich (Milan, Italy) and Merck KGaA (Darmstadt, Germany). 20-Hydroxyecdysone, 2-deoxy-20-hydroxyecdysone, and shaftoside, obtained from the Institute of the Chemistry of Plant Substances (Tashkent, Uzbekistan), were used in this study with purities > 99%. Cell culture media (DMEM), supplements, dimethylsulfoxide (DMSO) were obtained from Gibco (Waltham, MA, USA). Renilla luciferase and XtremeGENE 9 reagent were purchased from Addgene (Cambridge, MA, USA) and Roche Holding (Bazel, Switzerland), respectively.

### 2.2. Plant Material

The aerial parts (flowers, leaves, and stems) of *S. viridiflora* were collected from the botanical field of the Institute of the Chemistry of Plant Substances (Tashkent, Uzbekistan). The taxonomic authentication was accomplished by Dr. A. Nigmatullaev of the Department of Herbal Plants of the ICPS. The voucher specimen of the plant was deposited in the departmental herbarium under the code 2017/087. The plant material was air-dried and powdered before use.

### 2.3. Preparation of Extract for Bioassays

The aerial parts of *S. viridiflora* were washed gently and left to dry naturally at room temperature. The dried plant material was ground to a fine powder with a Waring blender. After grinding, 50–100 g of the plant material was extracted with 200–500 mL of methanol. The extraction process by maceration was carried out by immersing the plant material in methanol for one day. The solvent was evaporated in a rotary vacuum evaporator at 40 °C. The obtained extract was then kept in a refrigerator until further use.

### 2.4. Preparation of Extract for UHPLC-MS Measurements

A total of 10 mg of powdered plant material was accurately weighed and placed into a tube with a cover, and 5 mL methanol solvent was added. After 15 min of ultrasonication at 50 °C, the extract was centrifuged at 14,000 g for 10 min to remove debris. After centrifugation, the supernatant was transferred to vials (1 mL) and filtered by a poly-tetraflouroethylene filter with a pore size 0.45 μm. This solution has been used for the UHPLC-MS measurements.

### 2.5. UHPLC-QTOF-MS/MS Analysis

Metabolites present in the methanol extract of *S. viridiflora* were identified using an advanced analytical technique, UHPLC-QTOF-MS/MS. The extract was injected into an Acquity-UPLC (Waters Inc., Milford, MA, USA) and separated on a Nucleoshell RP18 (150 mm × 2 mm × 2.7 µm; Macherey & Nagel, Düren, Germany) at 40 °C. The sample volume injected was set at 5 μL. The ESI mass spectra were acquired in positive and negative ion electrospray ionization mode by scanning over the *m*/*z* range 100–1200. The mobile phase consisted of 0.3 mM ammonium formate with 0.7 mM formic acid in water (A) and acetonitrile (B). The column flow was set at 0.3 mL/min, the autosampler temperature was 4 °C. Data interpretation was carried out using Sciex PeakView 2.1 software and ACD/MS Fragmenter (ACD/Lab, Toronto, ON, Canada). The putative known and unknown compounds were annotated by the Human Metabolome Database, MassBank Spectral Database, METLIN Metabolomics Database, as well as by comparison with standard compounds.

### 2.6. Cell Cultures

The cytotoxic activity of the samples was screened against the human triple-negative breast cancer BT-20 (HTB-19), HCC1395 (ATCC^®^CRL-2324), MDA-MB-231 (CRM-HTB-26), and HEK293 (CRL-1573) embryonic kidney cell lines. The cells were cultured in Dulbecco’s modified eagle medium (DMEM) supplemented with 4.5 g/L glutamine and D-glucose, 10% fetal bovine serum (FBS), and a 1% penicillin/streptomycin mixture and incubated at 37 °C under a 5% CO_2_ humidified atmosphere. Cell passage and seeding performed after washing the adherent cells with PBS and detaching them using a Trypsin solution. The cells were detached and resuspended at 160,000 cells/mL and added into each well of a transparent 384-well plate in the final volume of 25 µL/well (4000 cells per well). The cells were maintained in DMEM containing 10% FBS at 37°C and 5% CO_2_ overnight. The next day, the 384-well plate was examined under an inverted microscope to identify cell seeding errors, growth characteristics, morphology and equal distribution. Then, the medium in each well was removed using a washer–dispenser (Biotek FX, Beersel, Belgium) and replaced by 40 μL of the fresh medium containing the indicated concentrations of compounds and incubated for 72 h.

### 2.7. Sample Preparation for Bioassays and Treatment of Cells

The 250 µg/mL concentrations of methanol extract and 20 mM concentrations of individual compounds were prepared as a stock solution in DMSO (Sigma-Aldrich Chemie GmbH, Taufkirchen, Germany) prior to use. A complete medium was used to prepare this solution. The samples were further serially diluted in DMSO into eleven different concentrations and then added to the complete cell culture medium so as to attain the final concentrations ranging from 250 to 8.6 µg/mL (250, 178.6, 127.6, 91.1, 65.1, 46.5, 33.2, 23.7, 16.9, 12.1, and 8.6 µg/mL) for the extract and 20 to 1.45 µM (20, 15.4, 11.8, 9.1, 7.0, 5.4, 4.1, 3.2, 2.5, 1.9, and 1.5 µM) for the individual compounds in 384-well plates, which were added in four replicates. A 40 µL sample containing medium from each concentration was dispensed into each well. The concentration of the solvent, DMSO, was equal in all the wells and was fixed at 0.05% in the medium. As a negative control, cells were treated with DMEM containing DMSO. Docetaxel (concentration of 0.02 µM to 6.9 nM) is used as a positive control.

### 2.8. MTT Assay

The cytotoxicity of the samples was determined in triplicate using the 3-(4,5-dimethylthiazol-2-yl)-2,5-diphenyltetrazolium bromide (MTT) cell viability assay. The next day, the medium of the 384-well plates treated with samples was removed. Then, 25 µL of MTT solution (0.5 mg MTT in 1 mL PBS) was added to each well and incubated at 37 °C for 2 h. After incubation with MTT for 2 h, the solution was removed from the wells by the washer–dispenser, and the formed formazan crystals were then dissolved in 25 µL of DMSO. After a further 10 min of incubation at room temperature, the samples were mixed briefly, and the absorbance was detected at 510 nm with a Tecan Infinite 200 Pro Reader (Tecan Group Ltd., Männedorf, Switzerland). The cell viability rate (%) was calculated by the following formula:

Cell viability rate (%) = ((OD of treated cells − OD of media (blank)/(OD of control cells − OD of media (blank)) × 100%

### 2.9. TOPFlash Assay

The TOPFlash assay was performed as described [12,13]. TNBC BT-20 cell line stably transfected with TopFlash reporter plasmid was seeded at 450,000 cells/mL in a white opaque 384-well plate in the final volume of 25 μL. The cells were maintained and incubated at 37 °C with 5% CO_2_ overnight for attachment. Subsequently, they were transfected by a plasmid encoding Renilla luciferase under the CMV promoter using 12 μg/mL of DNA and 40 μL/mL XtremeGENE 9 reagent as described in the manufacturer’s protocol. The next day, the medium in each well was replaced with a 20 μL fresh medium containing Wnt3a (2.5 μg/mL) and compound dilution. Compound dilutions were prepared by serial dilution in DMSO and diluted with the amount of medium necessary to obtain their final concentrations indicated on the figures and tables and maintain a concentration of DMSO of 0.05% in all assay points. Wnt3a was added after 1 h of preincubation with compound dilution. After overnight incubation, the supernatant in each well was removed by the washer–dispenser, and the luciferase activity was measured as described [12,13]. Briefly, the culture medium was completely removed from all wells of the plate. Next, the luciferase activity of the firefly and Renilla luciferases was detected sequentially in individual wells of a 384-well plate through the injection of corresponding measurement solutions in Tecan Infinite 200 Pro multifunctional plate reader with an injection module [12].

### 2.10. Statistical Analysis

The experiments were carried out in four replicates. Continuous variables were presented as the mean ± SD. The IC_50_ was determined as the drug concentration which resulted in a 50% reduction in cell viability or the inhibition of biological activity. The level of significance was set at *p* < 0.05. The IC_50_ (inhibitory concentration which caused 50% inhibition) was estimated using the linear regression method of plots of the percent of cell viability against the concentration of the tested compounds using GraphPad Prism 8.0.1 software (San Diego, CA, USA).

## 3. Results and Discussion

### 3.1. UHPLC-HR-MS Profiling of Metabolites in a S. viridiflora Extract

As part of an ongoing study on secondary metabolites of the aerial parts of *S. viridiflora* metabolites [3,8,14,15,16], we performed, for the first time, qualitative chromatographic analyses of comprehensive compounds by means of UHPLC-ESI-MS/MS detection. UHPLC-MS/MS metabolite profiling of *S. viridiflora* revealed richness in diverse plant compounds. The base peak chromatograms of the methanol extract of *S. viridiflora* in both negative and positive ionization modes are displayed in Figure S1. The tentatively identified compounds are summarized in Table 1. Seventy-one compounds were tentatively identified based on the retention times, MS and MS/MS accurate masses, fragmentation patterns in MS/MS spectra, relative ion abundance, and comparison with reference standards and literature data. Compounds belonged to various classes, including flavonoid glycosides (19 compounds), triterpene glycosides (15), ecdysteroids (12), amino acids (10), phenolics (7), organic acids (3), sugar compounds (3), and polyols (2). In this study, all the compounds from *S. viridiflora* except metabolites **31**–**33**, **36**, **40**, **49**, **50**–**51**, **58**, and **65** [3,8,14,15,16] were annotated for the first time.

#### 3.1.1. Flavonoids

Flavonoids were detected as the most abundant class, represented by 19 peaks belonging to flavanone, flavone, and flavonol subclasses, as *C*- and *O*-glycosides. In addition to the derivatives of apigenin, quercetin, and luteolin, a flavonone hesperidin was also detected in the extract. The annotated flavonoids are summarized in Table 1. Some researchers reported that the *Silene* species are rich sources of flavonoids, especially the *C*-glycosides of the flavones apigenin and luteolin [17]. The most common apigenin *C*-monoglycosides found in the studied *Silene* species are vitexin, isovitexin, and a number of their derivatives; luteolin derivatives, as well as orientin and others, are found slightly less frequently [18]. Chemical investigations show that most of the studied species contain vicenins, isovitexin, orientin, isoorientin and their 8-*α*, 6-*α*, and 6-*β* isomers, isosaponarin, and vitexin. The MS/MS analysis led to the successful identification of the aglycone moieties. MS/MS was performed to assist in structural elucidation, where the nature of sugars could be revealed from the elimination of the sugar residue, i.e., 162 amu (hexose, glucose) and 146 amu (rhamnose) or 132 amu (pentose, arabinose). Flavones are represented mainly by apigenin derivatives, which are considered the active principles of *S. viridiflora*. In this plant, apigenin flavones accumulate as *C*-glycosides **23**–**24**, **26**, **29**–**30**, **34**, **38**–**39**, and **42**, except compound **26**. Based on its fragmentation pattern and according to the literature, compound **26** was tentatively identified as cosmosiin (apigenin 7-*O*-glucoside). Quercetin *O*-glycosides were represented by five peaks (**41**, **43**, **46**, **47**, **54**), while peaks **25**, **35**, **37** and **45** represented luteolin glycosides. The major metabolite **29** was eluted at 7.14 min and has a [M − H]^−^ ion at *m*/*z* 563.1406 and the molecular formula C_26_H_28_O_14_. In the MS/MS spectra of this compound, the diagnostic ions at *m*/*z* 473.1054, 443.0950, and 383.0759 were produced by neutral losses of C_3_H_6_O_3_ (90Da), C_4_H_8_O_4_ (120Da), C_2_H_4_O_2_ (60Da), and +C_4_H_8_O_4_ (120Da), indicative of a flavonoid *C*-glycoside. By comparing the MS/MS spectra with the literature data [19], metabolite **29** was identified as schaftoside. The MS spectrum interpretation allows for the annotation of hesperidin signals with *m*/*z* 611.1980 [M + H]^+^ and 628.2221 [M + NH_4_]^+^ in peak **53**. This flavonoid earlier was isolated from *S. alba*, *S. conoidea*, *S. compacta*, *S. dichotoma*, *S. italica*, *S. supine*, *S. vulgaris*, and *S. schimperiana* (Table 1) [20,21,22].

**Table 1:** Metabolites identified in *Silene viridiflora* using UHPLC-ESI-MS/MS in both negative and positive ionization modes.

N	Rt (min)	Tentatively Identified Metabolites	Average *m*/*z*	Reference *m*/*z*	Error (ppm)	Adduct Type	MS/MS Fragments	Formula	Resource	Reference	Class
**1**	0.77	Histidine	156.0769	156.0768	0.8	[M + H]^+^		C_6_H_9_N_3_O_2_	*S. colorata*, *S. dioica*	[23,24]	Amino acids
**2**	0.78	Arginine	175.1189	175.1195	−3.4	[M + H]^+^		C_6_H_14_N_4_O_2_	*S. colorata*, *S. dioica*	[23,24]	Amino acids
**3**	0.81	Glutamine	145.0622	145.0619	2.1	[M − H]^−^		C_5_H_10_N_2_O_3_	*S. colorata*	[23]	Amino acids
			147.0765	147.0764	0.4	[M + H]^+^					
**4**	0.83	Glutamic acid	146.0452	146.0459	−4.7	[M − H]^−^		C_5_H_9_NO_4_	*S. alba*, *S. colorata*, *S. dioica*	[23,24]	Amino acids
**5**	0.84	Glucaric acid	209.0312	209.0303	4.4	[M − H]^−^		C_6_H_10_O_8_			Sugar compounds
**6**	0.85	Pinitol	195.0858	195.0863	−2.3	[M + H]^+^		C_7_H_14_O_6_	*S. brahuica*, *S. ruscifolia*	[25,26]	Polyols
7 *	0.85	Sucrose	341.1069	341.1084	−4.3	[M − H]^−^	387.1133 [M + HCOO]^−^	C_12_H_22_O_11_	*S. vulgaris*, *S. nutans*, *S. noctiflora*, *S. ruscifolia*	[26,27]	Sugar compounds
			343.1213	343.1235	−6.5	[M + H]^+^	365.1044 [M + Na]^+^				
**8**	0.88	Trehalose	387.1130	387.1144	−3.6	[M + HCOO]^−^	729.2293 [2M + HCOO]^−^	C_12_H_22_O_11_	*S. ruscifolia*	[26]	Sugar compounds
			360.1493	360.1500	−2.0	[M + NH_4_]^+^					
**9**	0.86	Proline	114.0549	114.0549	0.2	[M − H]^−^		C_5_H_9_NO_2_	*S. colorata*	[23]	Amino acids
			116.0709	116.0706	2.8	[M + H]^+^					
**10**	0.86	Norvaline	118.0859	118.0863	−3.6	[M + H]^+^		C_5_H_11_NO_2_			Amino acids
**11**	0.87	Threonine	120.0663	120.0661	1.7	[M + H]^+^		C_4_H_9_NO_3_	*S. colorata*, *S. dioica*	[23,24]	Amino acids
12 *	0.89	Quinic acid	191.0567	191.05611	3.1	[M − H]^−^		C_7_H_12_O_6_	*S. alba*, *S. conoidea*, *S. compacta*, *S. dichotoma*, *S. italica*, *S. supine*, *S. vulgaris*	[20,22]	Polyols
			193.07133	193.0707	3.3	[M + H]^+^					
**13**	0.89	Stachydrine	144.1017	144.1019	−1.2	[M + H]^+^		C_7_H_13_NO_2_			Amino acids
**14**	0.91	Diglycolic acid	133.0144	133.0142	1.0	[M − H]^−^		C_4_H_6_O_5_			Organic acid
**15**	0.93	Tyrosine	182.0803	182.0812	−4.8	[M + NH_4_]^+^		C_9_H_11_NO_3_	*S. colorata*	[23]	Amino acids
**16**	0.94	Fumaric acid	115.0036	115.0037	−0.5	[M − H]^−^		C_4_H_4_O_4_			Organic acid
**17**	1.3	Citric acid	191.0193	191.0197	−2.1	[M − H]^−^		C_6_H_8_O_7_	*S. vulgaris*	[28]	Organic acid
**18**	6.34	Tryptophan	203.0815	203.0826	−5.4	[M − H]^−^		C_11_H_12_N_2_O_2_			Amino acids
			205.0978	205.0972	3.1	[M + H]^+^					
**19**	6.47 *	Ferulic acid	193.0497	193.0506	−4.6	[M − H]^−^		C_10_H_10_O_4_	*S. pratensis*, *S. schimperiana*	[21,29]	Phenolics
**20**	6.51	Salidroside	299.1121	299.1136	−5.1	[M − H]^−^	599.2322 [2M − H]^−^, 345.1189 [M + HCOO]^−^	C_14_H_20_O_7_			Phenolics
**21**	6.53	*p*-Coumaric acid	163.0395	163.0401	−3.4	[M − H]^−^		C_9_H_8_O_3_	*S. alba*, *S. conoidea*, *S. compacta*, *S. dichotoma*, *S. italica*, *S. supine*, *S. vulgaris*	[20,22]	Phenolics
			165.0532	165.0530	1.2	[M + H]^+^	147.0440 [M + H − H_2_O]^+^				
**22**	6.69	Chlorogenic acid	353.0874	353.0878	−1.3	[M − H]^−^		C_16_H_18_O_9_	*S. alba*, *S. dichotoma*, *S. italica*, *S. supine*, *S. vulgaris*, *S. albae*, *S. pendulae*, *S. compacta*	[20,22,30]	Phenolics
			355.1004	355.1000	1.0	[M + H]^+^	372.1285 [M + NH_4_]^+^, 377.0836 [M + Na]^+^				
**23**	6.71	Isovitexin 7,2″-di-*O*-glucoside	757.2169	757.2186	−2.3	[M + H]^+^	779.1987 [M + Na]^+^	C_33_H_40_O_20_			Flavonoid glycoside
			755.2010	755.2040	−4.0	[M − H]^−^					
**24**	6.88	Isosaponarin (isovitexin 4′-*O-β*-D-glucopyranoside)	593.1499	593.1512	−2.1	[M − H]^−^		C_27_H_30_O_15_	*S. armeria*, *S. bupleuroides*, *S. chlorifolia*, *S. compacta*, *S. cretacea*, *S. cubanensis*, *S. polaris*	[2,31]	Flavonoid glycoside
**25**	6.89	Luteolin 6-*C-β*-D-glucoside-8-*C-α*-L-arabinoside (carlinoside)	579.1355	579.1350	0.9	[M − H]^−^	563.1368, 557.2928, 511.2869, 447.1487, 401.1426, 387.1638, 355.0651	C_26_H_28_O_15_	*S. repens, S. sibirica*	[32,33]	Flavonoid glycoside
**26**	6.92	Cosmosiin (apigenin 7-*O*-glucoside)	433.1128	433.1129	−0.4	[M + H]^+^	455.0948 [M + Na]^+^	C_21_H_20_O_10_	*S. succulenta*	[34]	Flavonoid glycoside
			431.0981	431.0984	−0.7	[M − H]^−^					
**27**	7.06	Vanillic acid	167.0345	167.0350	−3.0	[M − H]^−^		C_8_H_8_O_4_	*S. schimperiana*	[21]	Phenolics
**28**	7.12	Caffeic acid	181.0505	181.0501	2.2	[M + H]^+^	163.0385	C_9_H_8_O_4_	*S. dichotoma*, *S. italica*, *S. schimperiana*, *S. albae*, *S. pendulae*	[21,22,30]	Phenolics
**29**	7.14	Schaftoside (apigenin 6-*C-β*-D -glucoside-8-*C-α*-L-arabinoside)	563.1406	563.1401	0.9	[M − H]^−^	511.2904, 473.1054, 443.0950, 431.1878, 383.0759	C_26_H_28_O_14_	*S. aprica*, *S. repens*, *S. schafta*, *S. nemoralis*, *S. caramanica*, *S. sendtneri*, *S. frivaldszkyana*, *S. paradoxa*, *S. chalcedonica*	[17,32]	Flavonoid glycoside
			565.1538	565.1552	−2.6	[M + H]^+^	587.1359 [M + Na]^+^, 547.1468 [M + H − H_2_O]^+^				
**30**	7.21	Isovitexin 7-*O*-glucoside-2′’-*O*-rhamnoside	739.2094	739.2091	0.4	[M − H]^−^		C_33_H_40_O_19_	*S. pratensis*	[35]	Flavonoid glycoside
			741.2219	741.2236	−2.4	[M + H]^+^					
31 *	7.23	5,20,26-Trihydroxyecdysone (26-hydroxypolypodine B)	511.2913	511.2907	1.2	[M − H]^−^	557.2967 [M + HCOO]^−^, 447.0890	C_27_H_44_O_9_	*S. viridiflora*	[16]	Ecdysteroids
**32**	7.30	2-Deoxy-5,20,26-trihydroxyecdysone	519.2931	519.2928	0.7	[M + Na]^+^	479.30023, 461.29126, 443.27814	C_27_H_44_O_8_	*S. viridiflora*	[15]	Ecdysteroids
**33**	7.34	20-Hydroxyecdysone galactoside	641.3543	641.3537	−0.9	[M − H]^−^	687.3597 [M + HCOO]^−^, 613.2096, 563.1367, 461.1630	C_33_H_54_O_12_	*S. brachuica*, *S. viridiflora*	[7]	Ecdysteroids
**34**	7.40	Vicenin 2 (apigenin-6,8-di-*C*-glucopyranoside)	594.1589	594.1585	0.7	[M − H]^−^		C_27_H_30_O_15_	*S. boissieri*, *S. caramanica*, *S. chlorantha*, *S. colpophylla*, *S. commutata*, *S. cyri*, *S. foliosa*, *S. frivaldszkyana*, *S. graminifolia*, *S. jenissensis*, *S. italic*, *S. linicola*, *S. macrostyla*, *S. nemoralis*, *S. nutans*, *S. paradoxa*, *S. saxatilis*, *S. sendtneri*, *S. roemeri*, *S. wolgensis*	[2,36]	Flavonoid glycoside
			595.1638	595.1658	−3.3	[M + H]^+^					
**35**	7.43	Orientin (luteolin-8-*C-β*-D-glucoside)	447.0919	447.0933	−3.2	[M − H]^−^		C_21_H_20_O_11_	*S. armeria*, *S. boissieri*, *S. bupleuroides*, *S. chlorantha*, *S. chlorifolia*, *S. commutata*, *S. compacta*, *S. cretacea*, *S. cubanensis*, *S. cyri*, *S. foliosa*, *S. graminifolia*, *S. jenissensis*, *S. italica*, *S. linicola*, *S. macrostyla*, *S. nutans*, *S. polaris*, *S. saxatilis*, *S. vulgaris*, *S. wolgensis*	[2]	Flavonoid glycoside
			449.1081	449.1079	0.5	[M + H]^+^					
**36**	7.45	20,26-Dihydroxyecdysone	495.2963	495.2958	1.0	[M − H]^−^	541.3018 [M + HCOO]^−^, 439.1798, 393.1740	C_27_H_44_O_8_	*S. repens*, *S. viridiflora*	[17,37]	Ecdysteroids
**37**	7.49	Isoorientin (luteolin-6-*C-β*-D glucoside)	447.0925	447.0933	−1.8	[M − H]^−^		C_21_H_20_O_11_	*S. aprica*, *S. armeria*, *S. boissieri*, *S. bupleuroides*, *S. chlorantha*, *S. chlorifolia*, *S. commutata*, *S. compacta*, *S. cretacea*, *S. cubanensis*, *S. cyri*, *S. italic*, *S. littorea*, *S. foliosa*, *S. graminifolia*, *S. jenissensis*, *S. italica*, *S. macrostyla*, *S. nutans*, *S. polaris*, *S. saxatilis*, *S. viscariopsis*, *S. vulgaris*, *S. wolgensis*	[2]	Flavonoid glycoside
			449.1101	449.1078	5.2	[M + H]^+^	471.0893 [M + Na]^+^				
38 *	7.51	Saponarin (isovitexin 7-*O-β*-D-glucoside)	593.1498	593.1512	−2.3	[M − H]^−^		C_27_H_30_O_15_	*S. colorata*, *S. repens*, *S. nutans*	[1,38]	Flavonoid glycoside
			577.1530	577.1552	−3.9	[M + H]^+^	617.15063 [M + Na]^+^				
**39**	7.52	Vitexin-2″-*O*-rhamnoside (apigenin 8-*C-β*-D-glucoside-2″-*O*-rhamnoside)	577.1563	577.1557	1.0	[M − H]^−^	503.1104, 471.0893, 413.0855	C_27_H_30_O_14_	*S. nutans*	[38]	Flavonoid glycoside
			579.1704	579.1708	−0.7	[M + H]^+^	601.15365 [M + Na]^+^				
**40**	7.57	Integristerone A	541.3018	541.3013	1.0	[M + HCOO]^−^	447.0884	C_27_H_44_O_8_	*S. viridiflora*	[16]	Ecdysteroids
			519.2947	519.2928	3.7	[M + Na]^+^	479.30194, 461.29297, 443.27985				
41 *	7.58	Rutin (quercetin-3-*O*-rutinoside)	609.1440	609.1461	−3.5	[M − H]^−^		C_27_H_30_O_16_	*S. alba*, *S. conoidea*, *S. compacta*, *S. dichotoma*, *S. italica*, *S. supine*, *S. vulgaris*, *S. schimperiana*	[20,21,22]	Flavonoid glycoside
			611.1611	611.1607	0.7	[M + H]^+^					
**42**	7.73	Isovitexin (apigenin 6-*C-β*-D-glucopyranoside)	431.0984	431.0984	0.1	[M − H]^−^		C_21_H_20_O_10_	*S. alba*, *S. aprica*, *S. armeria*, *S. boissieri*, *S. brachuica*, *S. bupleuroides*, *S. chlorantha*, *S. chlorifolia*, *S. commutata*, *S. compacta*, *S. cretacea*, *S. cubanensis*, *S. cyri*, *S. diclinis*, *S. dioica*, *S. foliosa*, *S. graminifolia*, *S. jenissensis*, *S. italica*, *S. macrostyla*, *S. multifida*, *S. nutans*, *S. polaris*, *S. repens*, *S. supina*, *S. turgida*, *S. wolgensis*	[2,18]	Flavonoid glycoside
			433.1144	433.1129	3.3	[M + H]^+^	455.0926 [M + Na]^+^				
**43**	7.74	Quercetin-3-*O*-(6′’-*O*-malonyl)-*β*-glucoside	551.1030	551.1037	−1.3	[M + H]^+^	515.3002, 392.2085, 279.1587	C_24_H_22_O_15_			Flavonoid glycoside
**44**	7.79	26-Hydroxyecdysone	479.3014	479.3009	1.1	[M − H]^−^	1005.6133, 525.3069 [M + HCOO]^−^	C_27_H_44_O_7_	*S. repens*	[37]	Ecdysteroids
**45**	7.80	Diosmin (luteolin 4′-methyl ether-7-*O*-rutinoside)	607.1992	607.2000	−1.3	[M − H]^−^		C_28_H_32_O_15_	*S. succulent*, *S. schimperiana*	[21,34]	Flavonoid glycoside
			609.1842	609.1814	4.6	[M + H]^+^	631.1658 [M + Na]^+^				
**46**	7.82	Hyperoside (quercetin-3-*O-β*-D-galactoside)	463.0867	463.0873	−1.3	[M − H]^−^		C_21_H_20_O_12_	*S. albae*, *S. pendulae*, *S. compacta*	[20,30]	Flavonoid glycoside
**47**	7.83	Narcissin (3-methylquercetin-3-*O*-rutinoside)	623.1619	623.1618	0.2	[M − H]^−^		C_28_H_32_O_16_	*S. ruscifolia*	[26]	Flavonoid glycoside
**48**	7.83	Dihydroferulic acid	195.0654	195.0663	−4.7	[M − H]^−^		C_10_H_12_O_4_	*Gypsophila paniculata*	[39]	Phenolics
**49**	7.84	2-Deoxy-5,20,26-trihydroxyecdysone	495.2963	495.2958	1.0	[M − H]^−^	1037.6054, [2M + HCOO]^−^, 541.3018 [M + HCOO]^−^	C_27_H_44_O_8_	*S. viridiflora*	[15]	Ecdysteroids
			519.2918	519.2934	−3.1	[M + Na]^+^	479.2991, 461.2902, 443.2801				
50 *	7.91	20-Hydroxyecdysone	479.3014	479.3009	1.1	[M − H]^−^	525.3067 [M + HCOO]^−^	C_27_H_44_O_7_	*S. viridiflora*	[7]	Ecdysteroids
			481.3160	481.3160	−0.1	[M + H]^+^	503.2966 [M + Na]^+^, 463.3051 [M + H − H_2_O]^+^, 445.2938 [M + H − 2H_2_O]^+^, 427.2853 [M + H − 3H_2_O]^+^				
**51**	7.93	2-Deoxyintegristerone A	479.3014	479.3009	1.1	[M − H]^−^	525.3069 [M + HCOO]^−^, 441.1962, 369.0882	C_27_H_44_O_7_	*S. otitis*, *S. italica ssp. nemoralis*, *S. viridiflora*	[16]	Ecdysteroids
			498.3402	498.3425	−4.6	[M + NH_4_]^+^					
**52**	8.09	Vaccaroside B	1296.6219	1296.6225	−0.4	[M + NH_4_]^+^	1006.5222, 798.4625	C_60_H_94_O_29_	*Vaccaria segetalis*	[40]	Triterpenoids
**53**	8.12	Hesperidin	611.1980	611.1976	0.7	[M + H]^+^	628.2221 [M + NH_4_]^+^	C_28_H_34_O_15_	*S. alba*, *S. conoidea*, *S. compacta*, *S. dichotoma*, *S. italica*, *S. supine*, *S. vulgaris*, *S. schimperiana*	[20,21,22,23,24,25,26,27,28,29,30]	Flavonoid glycoside
54 *	8.34	Quercitrin (quercetin 3-*O-α*-L-rhamnoside)	449.1079	449.1078	0.2	[M + H]^+^		C_21_H_20_O_11_	*S. albae*, *S. pendulae*	[30]	Flavonoid glycoside
**55**	8.52	Armeroside E	1131.5229	1131.5223	0.5	[M − H]^−^	677.3533, 565.2568, 367.1237	C_54_H_84_O_25_	*S. armeria*	[41]	Triterpenoids
**56**	8.63	Silenegallisaponin J	1117.5436	1117.5431	0.5	[M − H]^−^	707.2547, 649.3389, 581.2617, 509.3094	C_54_H_86_O_24_	*S. gallica*	[42]	Triterpenoids
**57**	8.68	Sinocrassuloside II	1131.5229	1131.5223	0.5	[M − H]^−^	978.4760, 825.4244, 588.2588	C_54_H_84_O_25_	*S. viscidula*	[43]	Triterpenoids
58 *	8.70	2-Deoxy-20-hydroxyecdysone	509.3120	509.3114	1.1	[M + HCOO]^−^	463.3023 [M − H]^−^, 973.6228 [2M + HCOO]^−^	C_27_H_44_O_6_	*S. viridiflora*	[16]	Ecdysteroids
			465.3211	465.3216	−1.1	[M + H]^+^	487.3024 [M + Na]^+^, 447.3113 [M + H − H_2_O]^+^, 429.2997 [M + H − 2H_2_O]^+^, 411.2900 [M + H − 3H_2_O]^+^, 393.2787 [M + H − 4H_2_O]^+^, 355.2270, 331.2272, 287.2005				
**59**	8.71	Armeroside D	1149.5335	1149.53291	0.5	[M − H]^−^		C_54_H_86_O_26_	*S. armeria*	[41]	Triterpenoids
**60**	8.73	Makisterone C	553.3382	553.3377	1.0	[M + HCOO]^−^		C_29_H_48_O_7_			Ecdysteroids
			507.3327	507.3322	1.0	[M − H]^−^					
**61**	8.89	Armeroside G	1015.4755	1015.4750	0.5	[M − H]^−^		C_49_H_76_O_22_	*S. armeria*	[41]	Triterpenoids
**62**	8.97	Armeroside F	987.4806	987.4801	0.5	[M − H]^−^		C_48_H_76_O_21_	*S. armeria*, *S. viscidula*	[41,43]	Triterpenoids
**63**	9.40	3*β*,22*α*-Dihydroxyolean-12-en-23-al-28-oic acid 3-*O-α*-L-arabinopyranosyl-(1→3)-*β*-D-glucuronopyranoside	793.4016	793.4011	0.7	[M − H]^−^		C_41_H_62_O_15_	*S. odontopetala*	[44]	Triterpenoids
**64**	9.46	2-Deoxy-20-hydroxyecdysone-acetate	505.3171	505.31653	−4.1	[M − H]^−^		C_29_H_46_O_7_	*S. praemixta*, *S. wallichiana*	[45,46]	Ecdysteroids
65 *	10.12	Silviridoside	1115.5304	1115.5274	2.7	[M − H]^−^	774.3528, 580.2618, 557.2574	C_54_H_84_O_24_	*S. viridiflora*	[3]	Triterpenoids
			1117.5425	1117.5431	−0.5	[M + H]^+^	1134.5691, 253.1077				
**66**	10.45	3-O-*β*-D-Glycuronopyranosyl-quillaic acid 28-O-hexapyranosyl-pentapyranosyl-xylopyranosyl ester	1101.5123	1101.5118	0.5	[M − H]^−^	749.3395, 573.2545, 550.2504, 311.1664	C_53_H_82_O_24_			Triterpenoids
			1120.5534	1120.5540	−0.5	[M + NH_4_]^+^	971.4906, 680.4043, 571.2443				
**67**	10.55	3-*O*-[*β*-D-Glucopyranosyl-(1→2)-(*β*-D-xylopyranosyl-(1→3))-*β*-D-glucuronopyranosyl]-28-*O-β*-D-glucopyranosyl-3*β*-hydroxyolean-12-en-28-oic acid	1087.5331	1087.5325	0.5	[M − H]^−^	601.2628, 566.2651, 543.2607	C_53_H_84_O_23_	*Beta vulgaris*	[47,48]	Triterpenoids
			1106.5742	1106.5747	−0.5	[M + NH_4_]^+^	958.5082, 810.4453, 610.1826				
**68**	10.96	Acetyl-23-hydroxyolean-12-en-28-oic acid-dipentosyl-hexosyl-deoxy-pentoside	1085.5538	1085.5533	0.5	[M − H]^−^	965.4340, 666.2781, 565.2729, 482.2128	C_54_H_86_O_22_			Triterpenoids
			1104.5949	1104.5955	−0.5	[M + NH_4_]^+^	678.3821, 536.1646				
**69**	11.75	Quillaic acid-3-*O-β*-D-glucuronopyranoside	661.3593	661.3588	0.8	[M − H]^−^	535.3222, 279.1495, 185.0288	C_36_H_54_O_11_	*S. vulgaris*, *Psammosilene tunicoides*	[49,50]	Triterpenoids
			680.4004	680.4010	−0.9	[M + NH_4_]^+^	536.1642				
**70**	13.30	3*β*,22*α*-Dihydroxyolean-12-en-23-al-28-oic acid 3-*O-β*-D-glucuronopyranoside	707.3648	707.36427	−0.7	[M + HCOO]^−^	661.3556, 330.1734, 311.1661	C_36_H_54_O_11_	*S. odontopetala*	[44]	Triterpenoids
**71**	18.23	Oleanolic acid	455.3509	455.3525	−3.5	[M − H]^−^		C_30_H_48_O_3_	*S. succulenta*	[51]	Triterpenoids

*- Confirmed by comparison with corresponding reference standard.

#### 3.1.2. Triterpenes

More than 70 triterpenes have been isolated from 12 *Silene* species to date [52]. The characteristic features of these triterpenes are an aldehyde or carboxyl group at C-23, a carboxyl group at C-28, secondary alcoholic functions at C-16, and rarely at C-11 [2]—the methodology that permitted the tentative detection and successful characterization of 15 triterpene glycosides. The triterpene glycosides in the methanol extract of *S. viridiflora* were tentatively annotated as glycosides of gypsogenic acid (peak **52**), 16*α*-hydroxygypsogenic acid (**55**, **57**, **59**, **62**), caulophyllogenin (**56**), 3,16*α*-dihydroxy-3,4-secoolean-4(24),12-dien-23,28-dioic acid (**61**), 3*β*,22*α*-dihydroxyolean-12-en-23-al-28-oic acid (**63**, **70**), quillaic acid (**65**, **66**, **69**), 3*β*-hydroxyolean-12-en-28-oic acid (**67**, **71**), and oleanane type triterpene (**68**). Monitoring these MS data, the sequential loss of hexoses (Gal, Man), GlcA and/or pentoses (Xyl, Ara) in the presence of Glc as the dominant sugar were characteristic for triterpene peaks. The position of the sugar attachment could not be confirmed by MS analysis, but based on the preceding literature was assumed to connect to either the C-3 and/or C-28 positions of aglycone. The glycosylation pattern of *Silene* could be qualified by high relative levels of 28-Rha and 28-Fuc in gypsogenin and quillaic acid, 28-Glc in 16*α*-hydroxygypsogenic acid, and glucuronic acid in these three sapogenins [53]. Four 16*α*-hydroxygypsogenic acid (3*β*,16*α*-dihydroxyolean-12-en-23,28-dioic acid)-containing triterpene glycosides, **55**, **57**, **59**, and **62**, showing fragment ions of 16*α*-hydroxygypsogenic, were unequivocally identified following the data reported in the literature [54,55]. In the negative ion mode of UHPLC-MS, the oleanane type triterpenoid saponins mostly afford major [M − H]^−^ ions [50].

Recently, the first major peak **65** in TIC of *S. viridiflora* was isolated and through HR-MS and 1D and 2D NMR elucidated as silviridoside (3-*O*-*β*-D-galacturonopyranosyl-quillaic acid 28-*O-β*-D-glucopyranosyl-(1→2)-[*α*-L-rhamnopyranosyl-(1→3)]-*β*-D-fucopyranosyl ester) [3]. In this study, the second major peak **66** (R_t_ = 10.45 min) exhibited a parent ion [M − H]^−^ at *m*/*z* 1101.5223 with ion fragments at *m*/*z* 749.3395, 573.2545, 550.2504, and 311.1664. Under the positive ESI mode, the [M + NH_4_]^+^ adduct with *m*/*z* 1120.5534 was detected for this metabolite, with further fragmentation to produce mass fragments with *m*/*z* 971.4906 [M + H − Xyl]^+^, 680.4043, 571.2443. Thus, this compound could be assigned tentatively as 3-*O*-glycuronopyranosyl-quillaic acid 28-*O*-hexapyranosyl-pentapyranosyl-xylopyranosyl ester.

#### 3.1.3. Ecdysteroids

In this study, twelve known ecdysteroids were identified in the methanol extract of aerial parts of *S. viridiflora* by UHPLC-ESI-MS/MS (Table 1). These ecdysteroids, namely 5,20,26-trihydroxyecdysone (26-hydroxypolypodine B) (peak **31**), 2-deoxy-5,20,26-trihydroxyecdysone (**32**), 20-hydroxyecdysone galactoside (silenoside A or silenoside D) (**33**), 20,26-dihydroxyecdysone (**36**), integristerone A (**40**), 2-deoxy-5,20,26-trihydroxyecdysone (**49**), 20-hydroxyecdysone (**50**), 2-deoxyintegristerone A (**51**), and 2-deoxy-20-hydroxyecdysone (**58**), were previously isolated and characterized from *S. viridiflora* by CC and HPLC methods [8,14,15,16]. Also, three other ecdysteroids such as 26-hydroxyecdysone (**44**), makisterone C (**60**), and 2-deoxy-20-hydroxyecdysone-acetate (**65**) were annotated in *S. viridiflora* for the first time and were partially identified on the basis of their molecular weight and the fragmentation pattern. 26-Hydroxyecdysone (**44**, C_27_H_44_O_7_) was the major compound detected in *S. viridiflora* and was represented in the mass spectra with the signals at *m*/*z* 479.3014 [M − H]^−^, 525.3069 [M + HCOO]^−^ and 1005.6133 [2M + HCOO]^−^. Another major metabolite **51** in the positive ion mode, with [M + NH_4_]^+^ at *m*/*z* 498.3402, demonstrated a fragmentation pattern typical for 2-deoxyintegristerone A. In the negative ion mode, the spectrum of metabolite **51** fragments at *m*/*z* 525.3069 [M + HCOO]^−^, 479.3014 [M − H]^−^, 441.1962, 369.0882 was observed. Based on the comparison of the stacked experimental and reference mass spectra, this peak was identified as 2-deoxyintegristerone A (**51**). Similarly, 2-deoxy-20-hydroxyecdysone was identified as peak **58** based on the signals at *m*/*z* 463.3023 [M − H]^−^ and *m*/*z* 509.3120 and *m*/*z* 973.6228 corresponding to its adduct ions [M + HCOO]^−^ and [2M + HCOO]^−^ in negative ion mode. In the positive ion mode, metabolite **58** was observable as a pattern of ionic adducts—*m*/*z* 465.3211 [M + H]^+^ and 487.3024 [M + Na]^+^. Moreover, this pattern was accompanied with several water molecules, yielding the signals at *m*/*z* 447.3113 [M + H − H_2_O]^+^, 429.2997 [M + H − 2H_2_O]^+^, 411.2900 [M + H − 3H_2_O]^+^, and 393.2787 [M + H − 4H_2_O]^+^ (Figures S2 and S3). Most previous studies reporting on the LC-MS/MS analysis of ecdysteroids were conducted in the positive ion mode at the low (<10 eV) collision energy, which yields mass spectra showing a prominent loss of H_2_O molecules from polyhydroxylated molecular ions, that is, [M+X − (H_2_O)_n_]^+^, where X=H, Na, or K [56]. The presence of integristerone A (**40**), 20-hydroxyecdysone (**50**), and 2-deoxy-20-hydroxyecdysone (**58**) was confirmed by comparison with the reference spectra.

### 3.2. Anticancer Activities

Recent investigations of the *Silene* genus shows that its species have anticancer properties, including extracts of *S. firma, S. fortunei, S. succulenta* [57]. The total hydro-methanolic extract and its fractions of *S. succulenta* aerial parts against different cancer cell lines revealed that the highest activity was against breast carcinoma cell lines. The *n*-hexane fraction was a highly effective fraction against breast carcinoma cell lines (MCF-7) with IC_50_ = 15.5 mg/mL. After a bio-guided fractionation of the *n*-hexane fraction, two major compounds, cyclic glycolipids, were isolated and found to inhibit the proliferation of the MCF-7 cells at the IC_50_ of 21.5 µM and 13.1 µM [57].

In recent years, there has been growing interest in the potential use of ecdysteroids for the treatment of breast cancer. Martins et al. [58,59] demonstrated that semisynthetic ecdysteroid derivatives were able to inhibit the ABCB1 transporter and restore doxorubicin resistance in mammalian cancer cells expressing the human ABCB1 transporter. Studies have shown that ecdysteroids can inhibit the growth of breast cancer cells in vitro and in vivo. For example, a study by Romaniuk-Drapała et al. [60] showed that 20-hydroxyecdysone (ecdysterone) and ajugasterone C were able to inhibit the growth of triple-negative breast cancer MDA-MB-231 cells. Shuvalov et al. [61] found that 20-hydroxyecdysone strongly induces autophagy in a group of breast cancer cells (MCF-7, MDA-MB-231, and MDA-MB-468 cells). They also showed that 20-hydroxyecdysone exhibits a synergistic effect in combination with doxorubicin and induces cell death. However, this effect was observed in a high concentration range of 250–750 µM.

**Figure 1:**
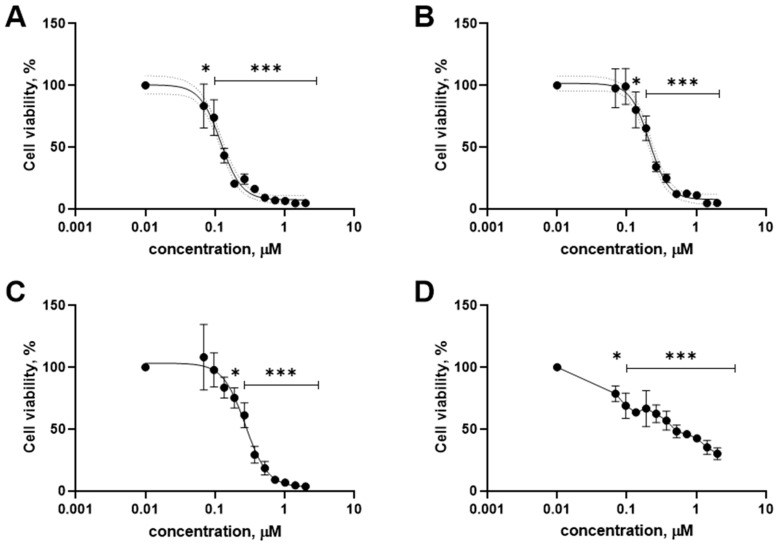
Inhibitory effect at various (2–0.069 µM) concentrations of 2-deoxy-20-hydroxyecdysone (**58**) on cell lines BT-20 (**A**), HCC1395 (**B**), MDA-MB-231 (**C**), and HEK293 (**D**) (MTT test). Data are represented as an average ±SEM from N = 3 independent experiments, performed in duplicate for each concentration (*n* = 2). Statistical significance was assessed by one-way ANOVA with multiple comparisons and is shown as * *p* < 0.05, *** *p* < 0.001 (for each point within the indicated span).

Zibareva et al. [62] reported that an ecdysteroid-containing *S. viridiflora* extract has antitumor effects in vivo. According to these previous studies, the real intention was to explore the cytotoxic compounds from the *S. viridiflora* aerial parts against cancer cells. Several ecdysteroids were previously isolated and characterized from *S. viridiflora* by CC and HPLC methods [8,14,15,16]. In this study, comprehensive metabolite profiling of *S. viridiflora* was carried out using the UHPLC-ESI-MS/MS method. In particular, we identified nine compounds such as quinic acid (**12**), ferulic acid (**19**), *p*-coumaric acid (**21**), schaftoside (**29**), isovitexin-7-*O*-glucoside (**38**), rutin (**41**), 20-hydroxyecdysone (**50**), 2-deoxy-20-hydroxyecdysone (**58**) and oleanolic acid (**71**) in the methanol extract of *S. viridiflora*. Except for the ecdysteroids **50** and **58** [7], the rest of the compounds were identified for the first time in *S. viridiflora*. The cytotoxic activity of some natural ecdysteroids (**72**–**76**) isolated from other species of *Silene, Ajuga*, and *Serratula* [2], along with their mechanism of action and structure–activity relationships was also evaluated. Furthermore, a methanol extract of *S. viridiflora* and compounds **12**, **19**, **21**, **29**, **38**, **41**, **50**, **58** and **71**–**76** was bioassayed by in vitro methods to determine the potential cytotoxic activity and for their ability to inhibit TNBC and healthy cells, which allowed a metabolite with the greatest inhibitory potential to be found (Table 2). The methanol extract showed significant cytotoxic effects towards BT-20, MDA-MB-231, HCC1395 and HEK293 cells at 10.1 ± 1.3, 10.4 ± 2.1, 9.27 ± 1.8 and >20 μg/mL with a viability of 50%. Among the tested samples, only 2-deoxy-20-hydroxyecdysone (**58**) was found to be the main inhibitor of cancer cells. This ecdysteroid exhibits strong cytotoxic activity against BT-20, HCC1395 and MDA-MB-231 cells, with an IC_50_ of 0.12 ± 0.006 μM, 0.21 ± 0.01 μM, and 0.53 ± 0.14 μM, respectively, while healthy HEK293 cells were inhibited at 0.27 ± 0.01 μM (Figure 1).

**Figure 2:**
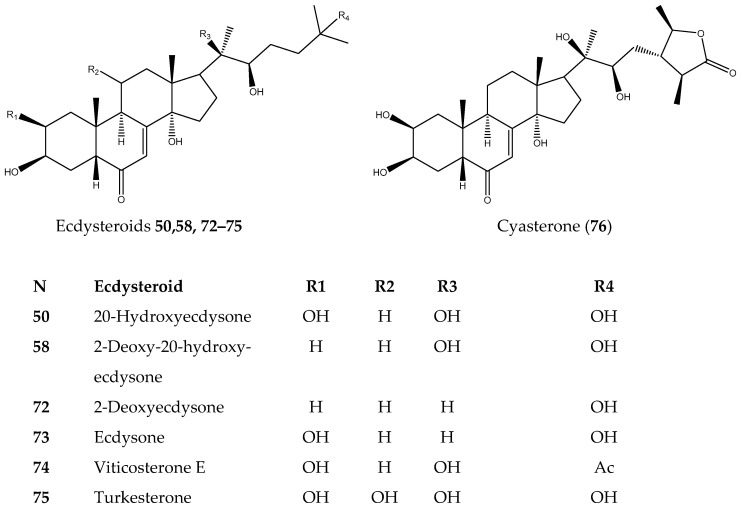
Structure of tested ecdysteroids.

**Figure 3:**
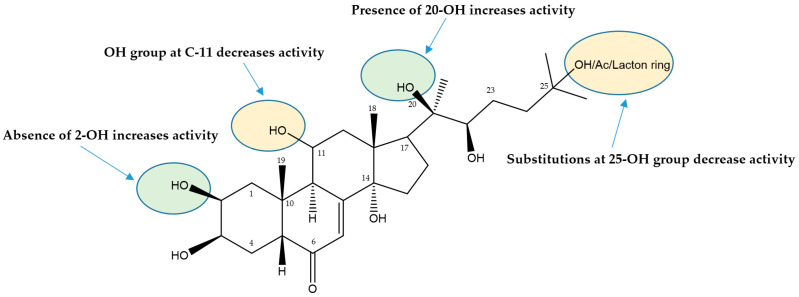
Summary of the SARs for the anti-proliferative activities of tested ecdysteroids.

A structure–activity relationship (SAR) study then determined the main structural features required to inhibit TNBC cells for the tested ecdysteroids **50**, **58**, and **72**–**76**. The inhibitory activity data presented in Table 2 led us to generate an initial SAR model in order to investigate the cytotoxic effect of the hydroxyl group on the steroid core, as well as the type of substituents (acetate and lactone ring at 25-OH) on the activity profile that could be explored (Figure 2 and Figure 3). Turkesterone (**75**), which possesses a hydroxyl group at C11 in the C ring, suggested that 11-OH also decreased the ecdysteroids’ cytotoxic activity. In contrast, the effects of substitutions of 25-OH in the side chain with an acetate group and lactone ring exemplified in viticosterone E (**74**) and cyasterone (**76**), if present, remain unclear due to activity above the highest concentrations tested of available derivatives. The structure–activity relationship inspected from the in vitro assay for ecdysteroids showed that the hydroxyl group present at position C-20 of the steroid core increases the ecdysteroids’ cytotoxicity against cancer cells. However, the absence of the hydroxyl group at position C-2 of the steroid core leads to a higher increase in the cytotoxicity of ecdysteroids (**58**) against TNBC cells by at least two orders of magnitude (Figure 3).

Martins et al. [63] studied the SAR of the apolar dioxolane derivatives of 20-hydroxyecdysone. The results showed that the substituted dioxolane ring at the 2,3 position is far more important for strong activity than the one at positions 20,22. The ecdysteroid derivative monosubstituted at position 2,3, was the only ecdysteroid derivative that was able to exert a stronger activity than the ecdysteroids with the 2,3; 20,22 diacetonide group.

Breast cancer in general and TNBC in particular are highly dependent on the oncogenic Wnt signaling for growth and progression [64,65], and a number of small molecules inhibiting TNBC cell proliferation through the blocking of their Wnt signaling has been identified [66,67,68,69]. In this study, we used the TOPFlash assay to assess the inhibitory effects of the methanol extract and compounds **12**, **19**, **21**, **29**, **38**, **41**, **50**, **58** and **71** of *S. viridiflora* on Wnt signaling. Since TNBC cells are known to be dependent on Wnt signaling, additional efforts were made to determine whether compounds derived from *S. viridiflora* could be active against this signaling cascade, although there was no reason to assume that this extract should have specific inhibitory activity against Wnt from the point of view of components or known activities. The TOPFlash assay is a versatile tool that can be used to study the Wnt signaling pathway in a variety of contexts, including drug screening, target validation, and mechanistic studies [70,71]. It is particularly useful for identifying inhibitors of the Wnt pathway, as these compounds will typically reduce Wnt-driven firefly luciferase activity in the assay. As a control of unspecific activity, e.g., cytotoxicity or the suppression of cell vitality and protein production, another luciferase, from *Renilla reniformis*, which is produced in a Wnt-independent manner and can be independently measured in the same wells, is used [72]. The obtained results showed that none of the tested samples of *S. viridiflora* inhibited Wnt signaling at a concentration of 20 µM (extract was tested at the 20 μg/mL concentration). Although 2-deoxy-20-hydroxyecdysone (**58**) has strong cytotoxic effect under the MTT test, the results suggested that it is not a Wnt signaling inhibitor and must act through another mechanism to prevent cancer cell proliferation.

## 4. Conclusions

In conclusion, this is the first comprehensive metabolite profiling of *S. viridiflora* using the UHPLC-ESI-MS/MS method. A total of 71 compounds were annotated and tentatively identified. The results have shown that flavonoids, triterpene glycosides, and ecdysteroids were the most abundant constituents in the species. The three major compounds schaftoside, 26-hydroxyecdysone, and silviridoside can be chosen as the markers for the assessment of the quality of *S. viridiflora* preparations. SAR studies for the ecdysteroids revealed that the absence of a hydroxyl group at C-2 in ring A and the presence of a hydroxyl group at C-20 on the side chain of ecdysteroids are important structural features for eliciting cytotoxic activity. These results suggest that the 2-deoxy-20-hydroxyecdysone can be employed/further developed as a chemotherapeutic drug candidate for the treatment of cancer. Future studies should include in vivo investigations to evaluate the effectiveness and safety of the compound.

## Figures and Tables

**Table 2:** In vitro cytotoxic activity of the methanol extract of *S. viridiflora* and compounds **12**, **19**, **21**, **29**, **38**, **41**, **50**, **58** and **71**–**76** against BT-20, HCC1395, MDA-MB-231, and HEK293 cell lines for exposure of 72 h (MTT test). The data are represented as an average ± SEM from N = 3 independent experiments, performed in duplicate for each concentration (*n* = 2).

N	Sample	BT-20	MDA-MB-231	HCC1395	HEK293
		IC_50_, µM			
**12**	Quinic acid	>20	>20	>20	>20
**19**	Ferulic acid	>20	>20	>20	>20
**21**	*p*-Coumaric acid	>20	>20	>20	>20
**29**	Schaftoside	>20	>20	>20	>20
**38**	Isovitexin-7-*O*-glucoside	>20	>20	>20	>20
**41**	Rutin	>20	>20	>20	>20
**50**	20-Hydroxyecdysone	>20	>20	>20	>20
**58**	2-Deoxy-20-hydroxyecdysone	0.12 ± 0.006	0.53 ± 0.14	0.21 ± 0.01	0.27 ± 0.01
**71**	Oleanolic acid	>20	>20	>20	>20
**72**	2-Deoxyecdysone	>20	>20	>20	>20
**73**	Ecdysone	>20	>20	>20	>20
**74**	Viticosterone E	>20	>20	>20	>20
**75**	Turkesterone	>20	>20	>20	>20
**76**	Cyasterone	>20	>20	>20	>20
	MeOH extract (μg/mL)	10.1 ± 1.3	10.4 ± 2.1	9.27 ± 1.8	>20
	Docetaxel (nM)	4.4 ± 0.0008	17.7 ± 0.0029	6.5 ± 0.0007	4.47 ± 0.0013

## Data Availability

Data are available upon request from the first author, N.Z.M.

## Supplementary Materials

The following supporting information can be downloaded at: https://www.mdpi.com/article/10.3390/biom14101285/s1, Figure S1: UHPLC-MS total ion chromatogram of the methanol extract obtained from *S. viridiflora* (A—negative ion mode, B—positive ion mode). Several peaks have been annotated in the chromatogram with the names of the metabolites shown in Table 1; Figure S2: (+) ESI-MS of 2-deoxy-20-hydroxyecdysone (**58**); Figure S3: Proposed MS/MS fragmentation of 2-deoxy-20-hydroxyecdysone (**58**) in the positive ion mode.
