# Supplementary material for: Chemical and Biological Characterization of Metabolites from Silene viridiflora Using Mass Spectrometric and Cell-Based Assays
Source: Biomolecules. 2024 Oct 11;14(10):1285. doi: 10.3390/biom14101285 (PMC11505650; doi:10.3390/biom14101285)
Supplement: Supplementary file 1 [file biomolecules-14-01285-s001.zip › biomolecules-3171449-supplementary.pdf]

Supporting Information

Chemical and biological characterization of metabolites from *Silene viridiflora* using mass spectrometric and cell-based assays

Nilufar Z. Mamadalieva [1-4,*], Alexey Koval [4], Maksud M. Dusmuratov [5], Hidayat Hussain [6], Vladimir L. Katanaev [4,*]

1 Institute of the Chemistry of Plant Substances, Uzbekistan Academy of Sciences, 100170 Tashkent, Mirzo Ulugbek Str. 77, Uzbekistan; nmamadalieva@yahoo.com (N.Z.M)
2 Tashkent Institute of Irrigation and Agricultural Mechanization Engineers, National Research University, 100000 Tashkent, Kori Niyazov Str. 39, Uzbekistan
3 Alfraganus University, Yuqori Qoraqamish Str. 2a, Tashkent 100190, Uzbekistan
4 Translational Research Center in Oncohaematology, Department of Cell Physiology and Metabolism, Faculty of Medicine, University of Geneva, 1211 Geneva, Switzerland; vladimir.katanaev@unige.ch (V.L.K); alexey.koval@unige.ch (A.K.)
5 Tashkent Pharmaceutical Institute, 100015 Tashkent, Oybek Str. 45, Uzbekistan; maqsudmansurovich17@gmail.com (M.M.D)
6 International Joint Laboratory of Medicinal Food Development and Health Products Creation, Biological Engineering Technology Innovation Center of Shandong Province, Heze Branch of Qilu University of Technology (Shandong Academy of Sciences), Heze 274000, China; hussainchem3@gmail.com (H.H)
* Correspondence: nmamadalieva@yahoo.com (N.Z.M); vladimir.katanaev@unige.ch (V.L.K)

A)

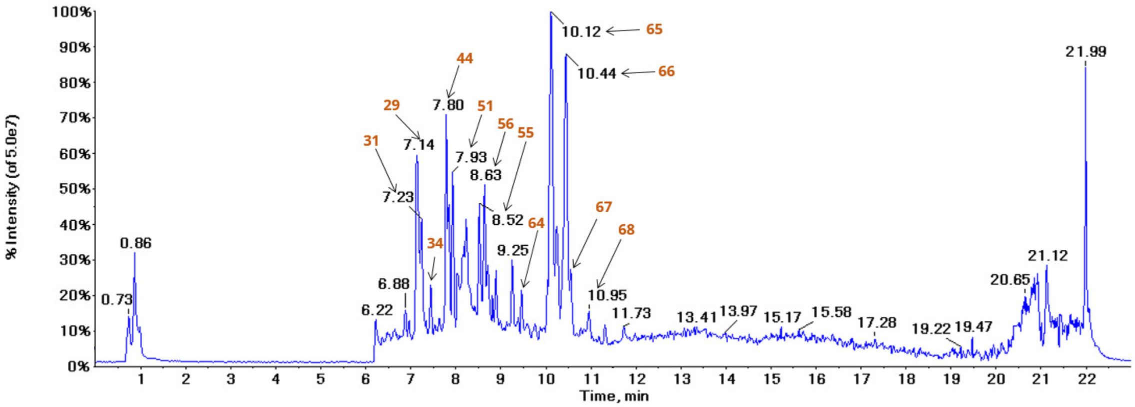

B)

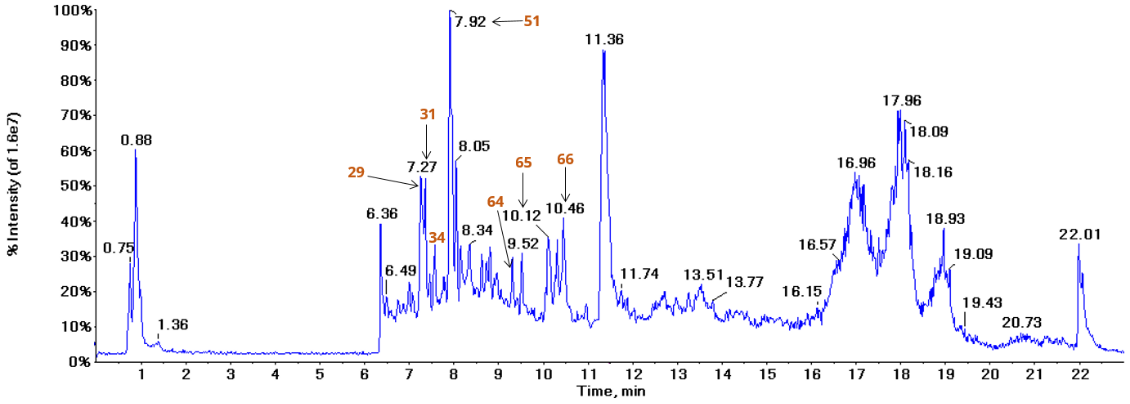

Figure S1. UHPLC-MS total ion chromatogram of the methanol extract obtained from *S. viridiflora* (A-negative ion mode, B- positive ion mode). Several peaks have been annotated in the chromatogram with the names of the metabolites shown in Table 1.

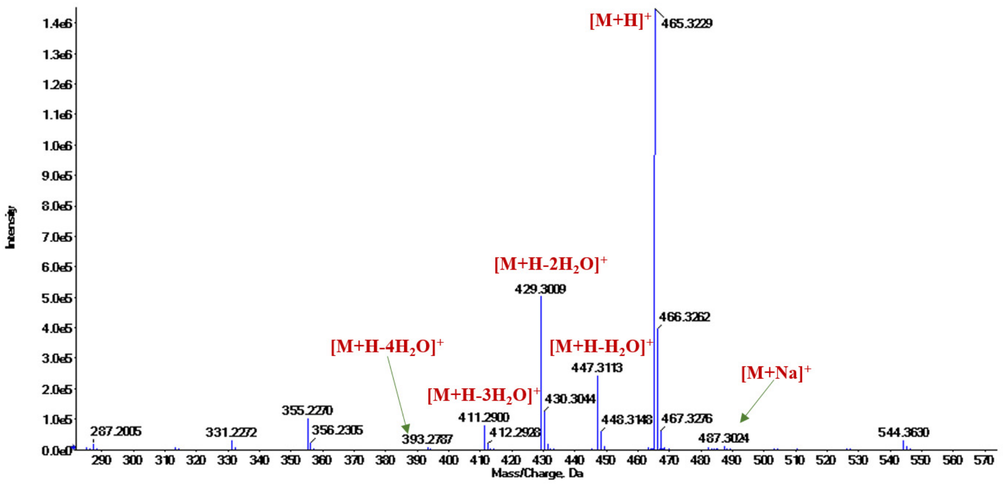

Figure S2. (+) ESI-MS of 2-deoxy-20-hydroxyecdysone (**58**)

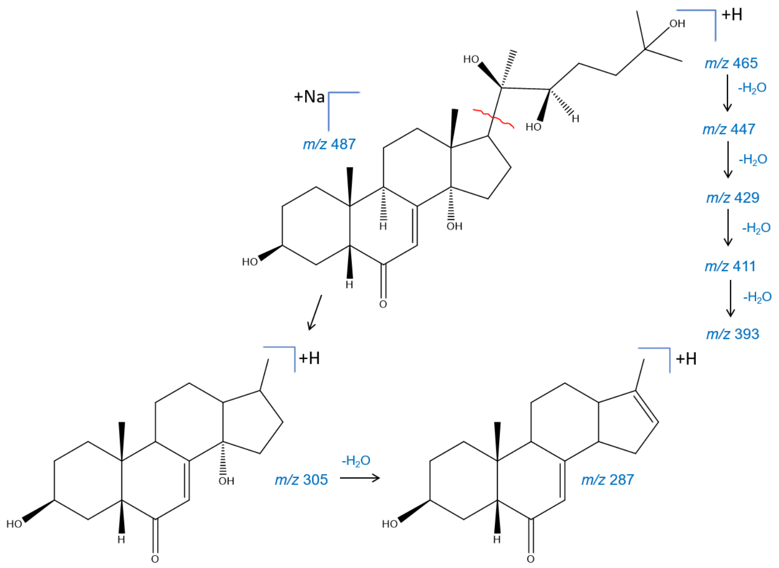

Figure S3. Proposed MS/MS fragmentation of 2-deoxy-20-hydroxyecdysone (**58**) in the positive ion mode
